# Microbial Genomic Consortia in Prostate Cancer: Mechanistic Signaling, the Gut–Prostate Axis, and Translational Perspectives

**DOI:** 10.3390/cancers18081219

**Published:** 2026-04-12

**Authors:** Eduardo Pérez-Campos Mayoral, Laura Pérez-Campos Mayoral, María Teresa Hernández-Huerta, Hector Alejandro Cabrera-Fuentes, Efrén Emmanuel Jarquín-González, Héctor Martínez-Ruiz, Margarito Martínez-Cruz, Carlos Romero-Diaz, Miriam Emily Avendaño-Villegas, Gabriel Mayoral-Andrade, Carlos Mauricio Lastre-Domínguez, Edgar Zenteno, María del Socorro Pina-Canseco, Primitivo Ismael Olivera González, Lucia Martínez-Martínez, Bernardo Rodrigo Santiago-Luna, Javier Vázquez-Pérez, Andrea Paola Cruz-Pérez, Diana Palmero-Alcántara, Tania Sinaí Santiago-Ramírez, Erico Briones-Guerash, Abelardo Augusto Ramírez-Davila, Juan de Dios Ruiz-Rosado, Eduardo Pérez-Campos

**Affiliations:** 1 Centro de Investigación Facultad de Medicina UNAM-UABJO, Facultad de Medicina y Cirugía, Universidad Autónoma “Benito Juárez” de Oaxaca, Oaxaca 68020, Mexico; eperezcampos.fmc@uabjo.mx (E.P.-C.M.); lperezcampos.fmc@uabjo.mx (L.P.-C.M.); hafuentes@iau.edu.sa (H.A.C.-F.); hmartinez.fmc@uabjo.mx (H.M.-R.); gmayoral.fmc@uabjo.mx (G.M.-A.); mpina.cat@uabjo.mx (M.d.S.P.-C.); primitiv52@gmail.com (P.I.O.G.); lumartin1969@yahoo.com.mx (L.M.-M.); dr_stgoluna@hotmail.com (B.R.S.-L.); javiervpch@gmail.com (J.V.-P.); cupa991107.fmc@uabjo.mx (A.P.C.-P.); dianalcantarapal@gmail.com (D.P.-A.); eribriogue@hotmail.com (E.B.-G.); aradavila@gmail.com (A.A.R.-D.); 2 Secretaría de Ciencia, Humanidades, Tecnología e Innovación (SECIHTI), Facultad de Medicina y Cirugía, Universidad Autónoma “Benito Juárez” de Oaxaca, Oaxaca 68020, Mexico; mthernandez@secihti.mx; 3 División de Estudios de Posgrado e Investigación, Tecnológico Nacional de México/Instituto Tecnológico de Tijuana, Tijuana 22414, Mexico; 4 R&D Group, Vice Presidency Scientific Research & Innovation, Imam Abdulrahman bin Faisal University (IAU), Dammam 31451, Saudi Arabia; 5 Dirección General de los Servicios de Salud de Oaxaca, Secretaria de Salud, Servicios de Salud de Oaxaca, Oaxaca 68000, Mexico; drefrenjg@icloud.com; 6 División de Estudios de Posgrado e Investigación, Tecnológico Nacional de México/Instituto Tecnológico de Oaxaca, Oaxaca 68030, Mexico; mcruz@itoaxaca.edu.mx (M.M.-C.); carlos.romero@itoaxaca.edu.mx (C.R.-D.); e_mily_3@hotmail.com (M.E.A.-V.); carlos.lastre@itoaxaca.edu.mx (C.M.L.-D.); 7 Facultad de Medicina, Universidad Nacional Autónoma de México, Ciudad de Mexico 04510, Mexico; ezenteno@unam.mx; 8 Dirección de la División de Investigación y Desarrollo Científico, Benemérita Universidad de Oaxaca, Oaxaca 68000, Mexico; ttanniaramirez@gmail.com; 9 Kidney and Urinary Tract Research Center, Abigail Wexner Research Institute, Nationwide Children’s Hospital, Columbus, OH 43215, USA; 10 Division of Nephrology and Hypertension, Nationwide Children’s Hospital, Columbus, OH 43205, USA; 11 Clinical Pathology Laboratory, “Eduardo Pérez Ortega”, Oaxaca 68000, Mexico

**Keywords:** prostate cancer, microbiome, gut–prostate axis, oncogenic signaling

## Abstract

**Background:** Prostate cancer (PCa) arises from complex interactions among host genetics, androgen signaling, and microbial communities. Emerging genomic evidence supports the presence of microbial consortia within prostate tissue, suggesting that microbial genes, metabolites, and host–microbe interactions may contribute to chronic inflammation, oncogenic signaling, and therapeutic resistance. **Methods:** We conducted a narrative review using targeted searches of PubMed and Google Scholar for studies published between 2020 and 2025, complemented by selected mechanistic reports published in March 2026. Human studies and experimental research providing mechanistic insights into prostate models were prioritized. Due to the heterogeneous methodologies, evidence was synthesized qualitatively, with an emphasis on genomic and signaling perspectives. **Results:** Low-biomass microbial DNA is consistently detected in prostate tissue. Proteomic analyses of *Corpora amylacea* suggest a “fossil record” of past infections through sequestered microbial DNA and antimicrobial proteins, potentially priming tissue for long-term carcinogenic processes, although contamination remains a key limitation. Recurrent bacterial and viral signals, including *Cutibacterium acnes*, *Escherichia coli*, *Pseudomonas*, *Acinetobacter*, human papillomavirus, Epstein–Barr virus, and cytomegalovirus, appear to converge on a restricted set of tumor-relevant pathways, including TLR–NF-κB, MAPK, PI3K/AKT/mTOR, cGAS–STING, and p53/pRb disruption. These interactions may promote cytokine production, oxidative stress, DNA damage, epithelial–mesenchymal transition, extracellular matrix remodeling, immune evasion, and resistance to therapy. The gut–prostate axis further links intestinal dysbiosis and microbial metabolites with systemic IGF-1 signaling and castration resistance. **Conclusions:** Microbial genomic consortia in the prostate and gut may shape inflammatory, metabolic, and immune networks that influence PCa initiation and progression. However, most available data remain correlative and are limited by low-biomass sampling, contamination risk, and heterogeneous study designs. Future research should prioritize rigorous contamination control, longitudinal and prostate-specific mechanistic studies, and integrated multi-omic approaches to clarify causality and identify actionable microbial targets for prevention, diagnosis, and therapy.

## Simple Summary

Prostate cancer pathogenesis involves complex interactions among host genetics, androgen signaling, and the local microbial environment. This narrative review, based on literature indexed in PubMed and Google Scholar up to March 2026, examines evidence that microbial genomic consortia may contribute to chronic inflammation, oncogenic signaling, disease progression, and treatment resistance. Microbial DNA has been consistently detected in prostate tissues, while *Corpora amylacea* may preserve a “fossil record” of past infections linked to chronic inflammation. Several bacterial and viral agents have been associated with convergent pathways involved in tumor biology, including NF-κB, MAPK, PI3K/AKT/mTOR, cGAS–STING, and p53/pRb disruption. The gut–prostate axis further links intestinal dysbiosis with systemic metabolic and immune signaling relevant to castration resistance. Although most findings remain correlative and are limited by the challenges of low-biomass microbiome research, current evidence supports a microbiome-informed model of prostate carcinogenesis with potential translational relevance for biomarkers and targeted interventions.

## 1. Introduction

The human holobiont encompasses an estimated 3.8 × 10^13^ microbial cells, establishing organ-specific niches across the genitourinary and gastrointestinal tracts [1]. In this context, microbial consortia represent functional, stable interactions between diverse species that modulate host homeostasis. It is now understood that a shift toward dysbiosis, or imbalance in the body’s microbial communities, can promote the development of inflammatory diseases and cancer [2].

Recent advances in microbiome research have shown that tissue-associated microbial communities can influence carcinogenesis through local inflammatory effects, systemic metabolic signaling, and immune modulation [3]. In several malignancies, including oral and colorectal cancers, pathobionts have been associated with the activation of convergent oncogenic pathways such as MAPK, NF-κB, and JAK–STAT, thereby shaping the tumor microenvironment and influencing disease progression [3,4]. These observations have strengthened the concept that tumor-associated microbiota may act not only as bystanders, but also as biologically relevant components of cancer ecosystems.

In the prostate, the historical view of sterility has been challenged by metagenomic, transcriptomic, and culture-independent studies detecting low-biomass microbial signatures in tissue, urine, and related compartments [5]. Persistent inflammation is recognized as a key feature of prostatic disease, ranging from benign hyperplasia to adenocarcinoma. Although the clinical relevance of inflammation is well established, its proximal triggers remain incompletely understood. Current evidence suggests that intraprostatic pathobionts and the systemic consequences of intestinal dysbiosis may provide chronic antigenic and metabolic stimuli that sustain a pro-inflammatory and tumor-permissive microenvironment [6].

Emerging studies further suggest that the prostate microbiota may co-evolve with tumor development, shifting from commensal or low-abundance states toward dysbiotic configurations associated with immune remodeling, altered metabolism, and potential effects on therapeutic response [6,7]. Candidate microorganisms such as *Cutibacterium acnes* (*C. acnes*), *Escherichia coli* (*E. coli*), *Pseudomonas aeruginosa*, *Mycoplasma* spp., and selected oncogenic viruses have been implicated in these processes, although the strength of evidence varies substantially across taxa and experimental models [5,8,9,10,11]. By synthesizing androgens de novo, metabolizing therapeutic agents, and altering host immune signaling, these microorganisms directly attenuate the clinical efficacy of standard interventions, notably Androgen Deprivation Therapy (ADT) [12,13,14,15,16].

**Figure 1:**
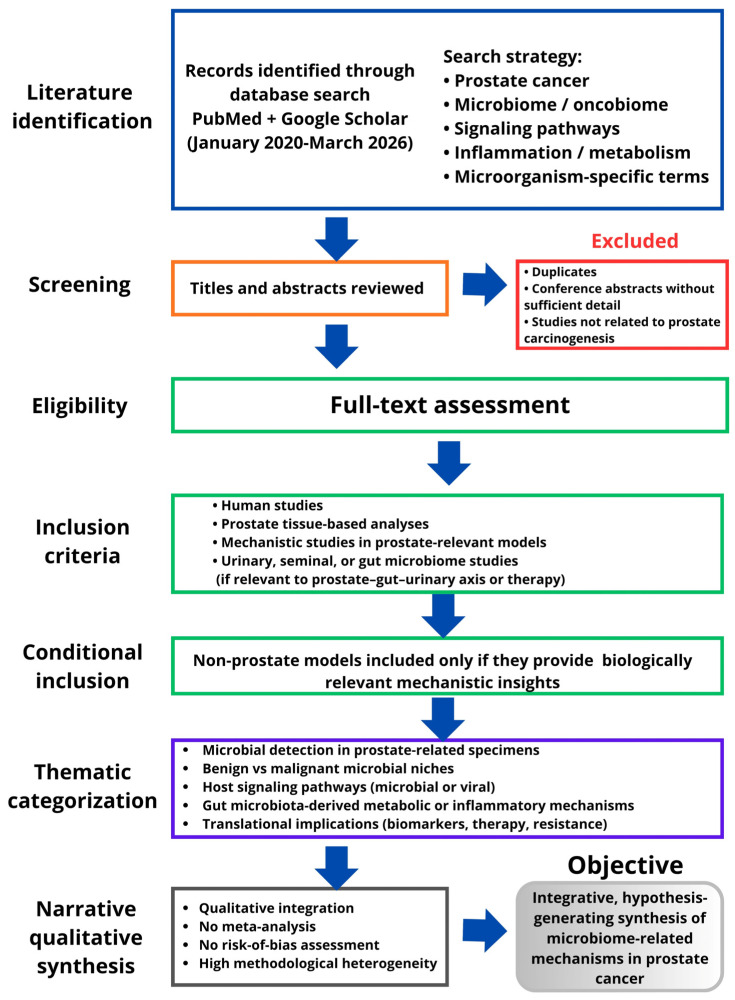
Thematic workflow for narrative literature identification and selection.

In this context, this review examines the hypothesis that microbial genomic consortia within the prostate may contribute to carcinogenesis by shaping inflammatory, metabolic, and immune signaling networks. To address this question, we conducted a targeted narrative review using PubMed and Google Scholar to identify studies published between January 2020 and March 2026 (Figure 1). Search terms combined prostate cancer-related concepts with microbiome, oncobiome, signaling, inflammation, metabolism, and microorganism-specific keywords. Priority was given to human studies, prostate tissue-based analyses, and mechanistic studies in prostate-relevant models, while urinary, seminal, and gut microbiome studies were included when relevant to the prostate–gut–urinary axis or treatment response. Articles were selected according to their relevance to microbial detection in prostate-related specimens, benign-versus-malignant microbial niches, convergent host signaling pathways, gut microbiota-derived metabolic and inflammatory mechanisms, and translational implications for biomarkers and therapeutic response. Because of substantial heterogeneity in sample types, detection methods, contamination control, and experimental design, the evidence was synthesized qualitatively rather than quantitatively. This review was conceived as a narrative, hypothesis-generating synthesis rather than a formal systematic review; accordingly, no meta-analysis or structured risk-of-bias assessment was performed.

## 2. The Prostate as a Microbial Ecosystem: Evidence of Intraprostatic Consortia

Defining the intraprostatic microbiome remains technically demanding due to its inherently low biomass. In contrast to the high bacterial density of the gastrointestinal tract, the prostate harbors only trace microbial DNA, complicating the differentiation between resident species and transient urinary microbes such as *Lactobacillus*, *Gardnerella*, *Staphylococcus*, and *Streptococcus*. While the gland was once viewed as sterile outside of acute infection, 16S rRNA sequencing in asymptomatic men has consistently detected microbial signatures, supporting the existence of a resident, albeit sparse, prostatic community [17,18]. However, the risk of contamination from environmental or cutaneous sources necessitates rigorous procedural controls. The frequent identification of common contaminants, such as *Escherichia* and *Pseudomonas*, has fueled skepticism regarding the biological validity of these findings; nevertheless, unique microbial signatures persist in studies utilizing high-sensitivity sequencing and template-free controls [8].

The “intra-prostatic ductal reflux” hypothesis provides a plausible mechanical explanation for this colonization. Anatomical vulnerability in the peripheral zone allows turbulent urine flow to transport urethral microbiota, including *Lactobacillus*, *Streptococcus*, and *Gardnerella*, into the prostatic ducts. This process is often exacerbated by urinary dysbiosis, which increases the pathogen load available for reflux. Once established, these bacteria facilitate biofilm formation and chronic, chemically induced inflammation, contributing to the development of a pro-oncogenic niche [18].

Substantial evidence for a historical microbial presence within the prostate is provided by the study of *Corpora amylacea* (CA), laminated hyaline concretions that increase in frequency with age. While historically regarded as inert physiological debris, recent research suggests these structures function as biomarkers of past infections. Proteomic and microscopic analyses indicate that CA act as “wasteosomes,” sequestering microbial products and host antimicrobial proteins, including S100A8/A9 and beta-2 microglobulin [19]. The successful extraction of microbial DNA and the identification of bacterial and fungal proteins within their concentric layers support the “Fossil Record” hypothesis: CA represent molecular evidence of prior infections even in the absence of a culturable microbiome. These historical insults may trigger chronic inflammatory cascades decades before a cancer diagnosis, serving as biological markers of the initial stimulus in a “hit-and-run” model of carcinogenesis [20,21]. The inflammatory and infectious landscape of the prostate includes primary candidates such as *C. acnes*, *Helicobacter pylori* (*H. pylori*), *Escherichia*, *Acinetobacter*, *Pseudomonas* spp., and *Shewanella* spp., each utilizing distinct molecular mechanisms [17].

## 3. Microbial Genomic Consortia and Tumor Ecology

Beyond the mere presence of individual microorganisms, prostate cancer appears to develop within a complex polymicrobial ecosystem in which diverse taxa coexist, interact, and collectively shape the tumor microenvironment. The oncogenic potential of the intraprostatic microbiota is best illustrated by the persistent colonization of *C. acnes* [10]. Multi-locus sequence typing (MLST) reveals a tissue-specific selection pressure; unlike cutaneous populations dominated by Type I strains, prostatic tissues are disproportionately enriched with Type II and Type III phylotypes [22]. These subtypes possess unique adaptations for survival within the hypoxic, lipid-rich prostatic microenvironment.

Additionally, *C. acnes* exhibits a capacity for intracellular persistence within macrophages, utilizing them as a stealth mechanism to evade immune clearance and establish chronic colonization [22]. *Shewanella* spp., typically aquatic bacteria, were found to be significantly over-abundant in malignant tissues. Significantly, tumors with high *Shewanella* loads exhibited a specific transcriptomic signature: the downregulation of Toll-like Receptor (TLR) signaling pathways and a depletion of dendritic cells [23]. This implies an immune evasion mechanism. By suppressing TLR signaling, *Shewanella* may create an immunosuppressive niche that protects the tumor from immune surveillance. The same study identified *Microbacterium* species as being significantly enriched in pathologically advanced T3 tumors (which have extended beyond the prostate capsule) compared to organ-confined T2 tumors [23]. This suggests that *Microbacterium* may either drive local invasion or thrive in the necrotic, hypoxic environment of a rapidly expanding tumor. Univariate Cox regression analyses have identified *Paenibacillus*, *Mycobacterium*, and *Streptococcus* as microbial markers associated with biochemical recurrence, with a rise in PSA after surgery [7]. Other studies have also reported anaerobic bacteria associated with cancer, such as *Fenollaria*, *Peptoniphilus*, *Porphyromonas*, *Anaerococcus*, and *Fusobacterium* [24]. Some studies have proposed *H. pylori* as a potential prostate carcinogen, whereas others interpret its detection as contamination or a secondary colonizer. This remains a controversial and unresolved hypothesis requiring independent validation. Notably, pan-pathogen microarray analyses have reported *H. pylori* signatures in more than 90% of prostate cancer specimens [25,26].

The intraprostatic oncobiota significantly contribute to carcinogenesis through direct genomic damage, modulation of inflammation, and subversion of host signaling pathways. Specifically, pks+ *E. coli* utilizes colibactin to induce double-strand DNA breaks and to modify Rho signaling via Cytotoxic Necrotizing Factor 1 (CNF1), a process that facilitates tumor invasion [27,28,29]. *H. pylori* has been proposed as a potential contributor to prostate carcinogenesis; however, most mechanistic data regarding CagA-mediated oncogenic signaling derive from gastric and other non-prostate models. In the context of the prostate, detection studies are heterogeneous, and direct evidence that CagA-driven mechanisms operate in situ remains lacking [30,31].

Enterotoxigenic *Bacteroides fragilis* has been shown in non-prostate models to cleave E-cadherin and activate β-catenin signaling, a mechanism that may be relevant to epithelial plasticity. In prostate cancer, however, this remains a biologically plausible extrapolation rather than a demonstrated mechanism [29,32]. Similarly, *Enterococcus* species induce oxidative stress and trigger NLRP3 inflammasome activation, establishing a pro-inflammatory microenvironment that promotes chronic inflammation and genomic instability. Emerging evidence further implicates *Mycobacterium* [33,34] and *Pseudomonas* [35] in accelerating cellular proliferation by constitutively activating the MAPK and KRAS signaling cascades.

Within this microbial niche, *Fusobacterium nucleatum* can impair antitumor immunity through Fap2–TIGIT interactions in other tumor contexts. Whether this immune-evasive mechanism operates in prostate cancer has not been directly established [36]. Collectively, this polymicrobial consortium establishes a permissive microenvironment where persistent antigenic stimulation and the systemic subversion of homeostatic checkpoints drive oncogenesis.

In oncogenesis caused by infectious pathogens, viral oncoproteins participate, potentially modulating cell proliferation by suppressing senescence or immune surveillance. Coinfection can accelerate transformation through mechanisms such as immunosuppression, chronic inflammation, and direct molecular communication, effectively overcoming host barriers. This multifactorial cooperation creates an environment conducive to genetic aberrations and malignant progression, for example, in *H. pylori* and EBV, EBV and malaria, and HTLV-1 and parasites. In prostate cancer, there is evidence [37]. For example, Cytomegalovirus (HCMV) immediate-early proteins IE1 and IE2 cooperate with adenovirus E1A to transform cells [38]. Also, in cervical carcinoma, HCMV and EBV are reported as cofactors that act together with HPV16 to enhance oncogenesis [39]. Given that diverse microbial consortia (on the order of dozens of bacterial genera) can coexist within a single prostate, their coexistence in its pathology is relevant [9].

The evidence implicating these pathogens in prostate carcinogenesis is, in many cases, incomplete. This results in an overall picture of limited, heterogeneous data, in some cases with clear, unresolved contradictions and reliance on extrapolation from non-prostate-cancer models. However, there are some cases with clear, albeit still partial, evidence. *C. acnes* has the most internally consistent body of work: Davidsson et al. found it four times more prevalent in cancerous prostates than in controls, with a fourfold increase in cancer odds after adjustment for confounders [40]; Fassi Fehri et al. demonstrated that *C. acnes* activates NF-κB and STAT3, induces IL-6 and IL-8 secretion, and enables anchorage-independent growth of prostate epithelial cells in vitro [41]; and Ashida et al. recently showed that *C. acnes* invades prostate epithelial cells, downregulates BRCA2, and impairs homologous recombination repair—a finding they termed “BRCAness” [42]. Yet even here, no prospective human study has demonstrated that *C. acnes* infection precedes and independently predicts cancer development, and the persistent problem of sample contamination by this ubiquitous skin commensal undermines confidence in detection rates across studies [11]. Finally, HCMV has attracted renewed interest since Samanta et al. detected HCMV proteins and nucleic acids in all 22 examined PIN and carcinoma lesions [43], and Classon et al. recently reported CMV infection in 70–92% of prostate tumors, with loss-of-function experiments showing that CMV promotes prostate cancer cell survival and proliferation, and that the CMV UL97 kinase inhibitor maribavir reduces tumor growth in xenotransplantation models [44]. These are provocative findings, but whether HCMV is causative or merely an opportunistic passenger in an immunologically permissive tumor microenvironment remains the central unresolved question [45].

Recent data indicate that prostate cancer and benign prostatic hyperplasia (BPH) develop in related but biologically distinct microbial niches. Tumor-based transcriptomic profiling shows that malignant prostate tissue contains a structured intratissue microbiome, with enrichment of genera such as *Shewanella* and other pathobionts compared with adjacent benign regions [46]. These shifts were associated with reduced Toll-like receptor signaling, altered focal adhesion, actin cytoskeleton, and extracellular matrix–receptor interaction pathways, as well as changes in immune-cell enrichment, consistent with a reprogrammed tumor microenvironment [46,47]. A systematic review and meta-analysis of genitourinary microbiomes found that specific bacterial taxa are more abundant in prostate cancer than in non-cancer controls, supporting the concept of a cancer-associated dysbiotic niche [10,48]. In contrast, the BPH milieu appears more closely linked to chronic, low-grade inflammation associated with alterations in both the local and gut microbiota. Recent reviews of the gut–prostate axis describe BPH-associated dysbiosis characterized by an increased Firmicutes/Bacteroidetes ratio, changes in *Prevotella*, *Ruminococcus*, and *Lactobacillus*, and enterotypes enriched in *Blautia*, *Bacteroides*, and *Streptococcus*; increased branched-chain fatty acid levels have also been associated with BPH [49,50,51]. Mendelian randomization analyses implicate *Escherichia–Shigella* in BPH and lower urinary tract symptom burden, supporting a potential inflammatory gut–prostate axis [52,53]. Tissue-based comparisons between BPH and prostate cancer document distinct microbial signatures, while available data suggest that chronic inflammation contributes to benign remodeling and that viral co-detection may be linked to malignancy-associated microbial patterns [10,46,54]. Collectively, these studies support the view that BPH arises within a hormonally modulated, microbiota-associated inflammatory niche that favors proliferative remodeling, whereas prostate cancer is characterized by a more deeply dysbiotic and immunosuppressed microenvironment. To further clarify these differences, the main features distinguishing microbial and microenvironmental niches in BPH and prostate cancer are summarized in Table 1.

## 4. Mechanistic Signaling Driven by the Prostate Oncobiome

**Figure 2:**
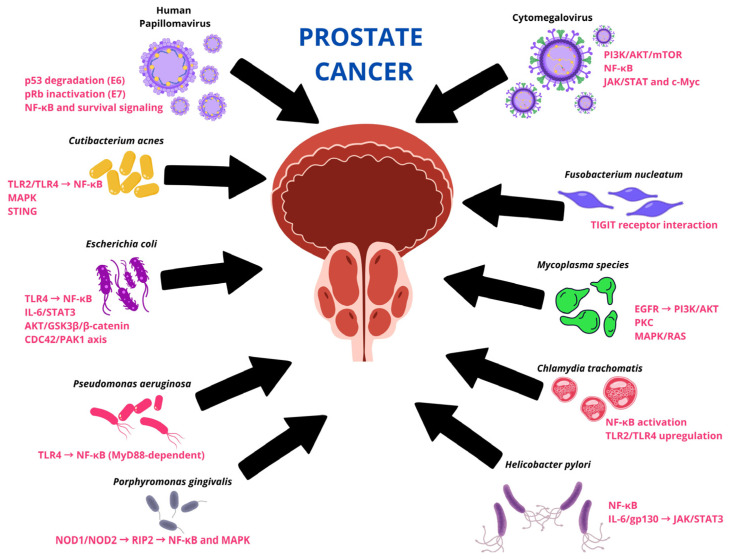
Signaling pathways that have been reported with different genera and species of the prostate cancer-related microbiome. Abbreviations: pRb, retinoblastoma protein; NF-κB, nuclear factor kappa B; TLR, Toll-like receptor; MAPK, mitogen-activated protein kinase; STING, stimulator of interferon genes; IL-6, interleukin 6; STAT3, signal transducer and activator of transcription 3; AKT, protein kinase B; GSK3β, glycogen synthase kinase 3 beta; CDC42, cell division cycle 42; PAK1, p21-activated kinase 1; MyD88, myeloid differentiation primary response 88; NOD1/NOD2, nucleotide-binding oligomerization domain-containing proteins 1 and 2; RIP2, receptor-interacting serine/threonine-protein kinase 2; PI3K, phosphoinositide 3-kinase; mTOR, mechanistic target of rapamycin; JAK, Janus kinase; c-Myc, MYC proto-oncogene; EGFR, epidermal growth factor receptor; PKC, protein kinase C; RAS, rat sarcoma virus oncogene homolog; gp130, glycoprotein 130; TIGIT, T cell immunoreceptor with Ig and ITIM domains.

While increasing evidence supports the presence of diverse microbial consortia within the prostate, their biological relevance is best understood through the molecular pathways they influence. Rather than acting as isolated infectious agents, bacteria and viruses within the prostatic microenvironment appear to converge on a limited set of host signaling networks that regulate inflammation, proliferation, immune evasion, and genomic stability (Figure 2). These interactions link chronic microbial exposure to key oncogenic processes and provide a mechanistic framework connecting the prostate oncobiome with tumor initiation and progression. The following section synthesizes current evidence on the principal signaling pathways activated by microbial components in prostate epithelial and stromal cells. In vitro studies indicate that *C. acnes* can induce IL-6 and CXCL8 (IL-8) secretion in prostate epithelial cells. However, within the prostate tumor microenvironment, these cytokines are also substantially amplified by stromal and immune compartments, particularly cancer-associated fibroblasts and myeloid cells. Therefore, *C. acnes* should be interpreted as a potential trigger of localized inflammatory signaling rather than the sole cellular source of IL-6 and IL-8 in prostate cancer [68]. These cytokines are potent drivers of proliferation and angiogenesis. However, clinical translation has been mixed. *C. acnes* was present in prostate cancer (PCa) patients; there was no significant difference in serum levels of these cytokines compared to uninfected controls [7]. This distinction is important because stromal–epithelial crosstalk may sustain chronic NF-κB/STAT3 signaling even when direct epithelial infection is focal or transient, thereby magnifying the biological effect of microbial stimuli within the tumor niche [69,70,71]. Taken together, these findings suggest that *C. acnes*-associated inflammation is more likely to be localized than systemic, generating microscopic inflammatory niches within the prostate [11]. Nevertheless, there is still no evidence demonstrating a causal role for *C. acnes* in human prostate cancer [72]. Despite taxonomic diversity, most microorganisms converge on a restricted set of oncogenic signaling pathways.

Among the signaling pathways of microbial agents are WNT/β-catenin, NF-κB, TLRs, ERK, and interferon-stimulating genes (STING) [73]. The prostate cancer microenvironment hosts a complex ecosystem of microbes that activate specific signaling pathways through distinct molecular mechanisms. Evidence indicates that bacterial and viral components predominantly activate inflammatory and oncogenic pathways, including NF-κB, MAPK, PI3K/AKT, TLR, and p53/pRb inactivation cascades. The functional connectivity of the oncobiota is evidenced by the convergence of signaling pathways, including NF-κB, PI3K/AKT/mTOR, and cGAS-STING.

**Figure 3:**
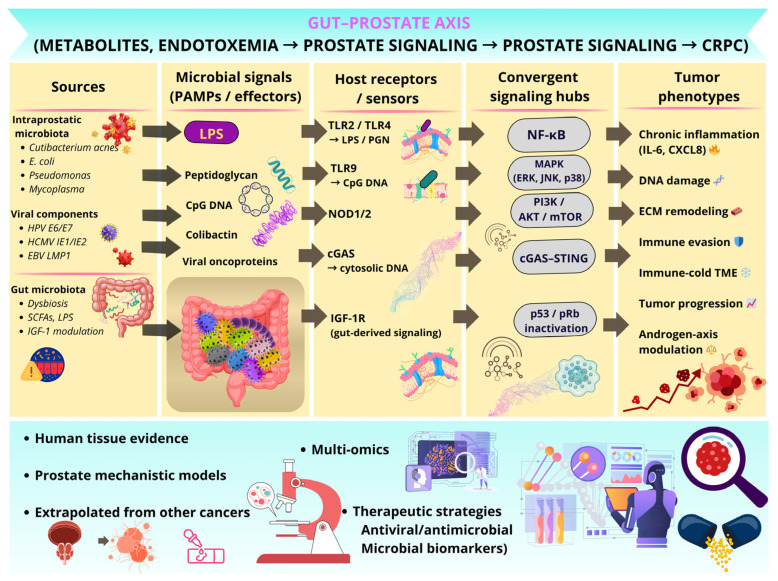
Integrated prostate oncobiome model: from microbial signals to tumor phenotypes across the gut–prostate axis. Abbreviations: CRPC, castration-resistant prostate cancer; PAMPs, pathogen-associated molecular patterns; LPS, lipopolysaccharide; PGN, peptidoglycan; TLR, Toll-like receptor; TLR2/4, Toll-like receptor 2/4; TLR9, Toll-like receptor 9; NOD1/2, nucleotide-binding oligomerization domain-containing proteins 1 and 2; cGAS, cyclic GMP–AMP synthase; STING, stimulator of interferon genes; IGF-1R, insulin-like growth factor 1 receptor; NF-κB, nuclear factor kappa B; MAPK, mitogen-activated protein kinase; ERK, extracellular signal-regulated kinase; JNK, c-Jun N-terminal kinase; PI3K, phosphoinositide 3-kinase; AKT, protein kinase B; mTOR, mechanistic target of rapamycin; p53, tumor protein p53; pRb, retinoblastoma protein; IL-6, interleukin 6; CXCL8, C-X-C motif chemokine ligand 8; ECM, extracellular matrix; TME, tumor microenvironment; SCFAs, short-chain fatty acids; HPV, human papillomavirus; HCMV, human cytomegalovirus; EBV, Epstein–Barr virus.

For example, in *C. acnes* (formerly *Propionibacterium acnes*), key signaling pathways include TLR2/TLR4–NF-κB, MAPK, and cGAS–STING. Peptidoglycan (PGN), the main structural component of the bacterial cell wall, acts as the principal pathogen-associated molecular pattern (PAMP) recognized by host pattern-recognition receptors. PGN binds to TLR2 and TLR4 on prostate epithelial cells, triggering MyD88-dependent signaling cascades that lead to NF-κB nuclear translocation and activation of MAPK pathways. This results in the production of proinflammatory cytokines, particularly IL-6 and IL-8, promoting a chronic inflammatory microenvironment that may contribute to prostate carcinogenesis [74]. Recent peptide array analysis revealed seven specific peptides (A14, A15, B1, B2, B3, C1, C3) within CAMP1 that bind TLR-2, located on one structural side, forming a binding pocket [75]. Given the complexity and convergence of microbe-driven signaling pathways, an integrative model is presented to synthesize current evidence across molecular and systemic levels (Figure 3). This schematic connects microbial signals, host sensing mechanisms, and downstream oncogenic processes with tumor phenotypes and the gut–prostate axis.

TLRs may play a dual role in prostate cancer. On the one hand, they are essential for clearing infections; on the other, their chronic stimulation could promote carcinogenesis. Expression of TLR4 is often upregulated in PCa cells and is associated with poor survival. It senses lipopolysaccharides (LPS) from Gram-negative gut or urinary bacteria. Activation of TLR4 on tumor cells can increase invasion and metastasis via STAT3 activation [76]. However, some studies report downregulation of TLR4 in high-grade tumors, potentially as a mechanism to evade immune detection [77]. TLR9 recognizes unmethylated CpG DNA motifs, a characteristic feature of bacterial genomes. Its expression is significantly increased in prostate cancer compared with benign prostatic hyperplasia and has been associated with higher Gleason scores, suggesting a link with more aggressive disease [78]. Under the ‘fossil record’ hypothesis, *Corpora amylacea* may function as a reservoir for microbial molecular patterns. Specifically, the sequestration of unmethylated CpG DNA within these concretions could provide a chronic stimulus for TLR9, sustaining a pro-inflammatory microenvironment long after the initial infection has subsided [79]. By dampening the “danger signal,” the tumor prevents the recruitment of cytotoxic T-cells and Dendritic Cells (DCs), maintaining an “immune cold” microenvironment that favors tumor growth [80]. The main prostate-associated microorganisms, their virulence factors, and the principal signaling pathways they activate are summarized in Table 2.

## 5. Viral Members of the Microbial Consortium

In addition to bacterial taxa, several viruses have been detected in prostate tissue and may contribute to tumor-associated signaling networks. Although their role in prostate carcinogenesis remains largely associative, current evidence suggests that viral species may act as modulators of proliferation, immune evasion, and genomic instability. For clarity, the main viral members are described individually below.

### 5.1. Human Papillomavirus (HPV)

Human Papillomavirus (HPV) is a well-established carcinogen in cervical and head-and-neck cancers [94]. In the prostate, high-risk HPV types, particularly HPV-16 and HPV-18, have been detected in malignant tissue, exosomes, and urine-derived samples, although prevalence varies across studies and detection methods [91,92,93,95]. HPV has been proposed to contribute to oncogenic processes mainly through the E6 and E7 oncoproteins, which promote p53 degradation and functional inactivation of pRb, respectively. Disruption of these key cell-cycle checkpoints may facilitate bypass of senescence, impaired DNA repair, and progressive genomic instability, thereby favoring malignant progression [96,97]. However, the strength of evidence remains limited. Most available studies are observational and demonstrate detection of viral DNA or proteins rather than direct causality [92,93,98]. In addition, heterogeneity in detection techniques and uncertainty regarding the biological significance of viral detection complicate interpretation [92,98]. Therefore, HPV should be regarded as an alleged contributor rather than a confirmed driver of prostate carcinogenesis [92].

### 5.2. Human Cytomegalovirus (HCMV)

Human cytomegalovirus (HCMV) has been detected in prostate intraepithelial neoplasia (PIN) lesions and carcinoma samples, with some studies reporting high prevalence rates in tumor tissues [43,44,99,100]. Unlike classical transforming viruses, HCMV is thought to act primarily as an oncomodulator [44,101]. Viral proteins such as IE1, IE2, UL38, UL133–UL138, and US28 can activate host pathways including PI3K/AKT/mTOR, NF-κB, and JAK/STAT, thereby promoting cell survival, proliferation, angiogenesis, and resistance to apoptosis [38,44,101,102]. Experimental studies further suggest that pharmacologic inhibition of viral kinases may reduce tumor growth, supporting a functional interaction between HCMV and tumor biology [44]. Despite these findings, the issue of causality remains unclear. The high prevalence of HCMV in tumor tissue may reflect opportunistic infection or viral persistence within an already permissive tumor microenvironment rather than a primary oncogenic role [99]. Thus, current evidence supports an oncomodulatory but not definitively causative role for HCMV in prostate cancer.

### 5.3. Epstein–Barr Virus (EBV)

Epstein–Barr virus (EBV) DNA and viral products have been identified in prostate cancer specimens, although detection rates vary substantially among studies [97,103,104]. The oncogenic effects of EBV are mainly mediated through latent viral proteins such as LMP1, LMP2, and EBNA1 [105,106]. These factors can activate NF-κB, MAPK, and PI3K/AKT signaling, inhibit apoptosis, enhance angiogenesis, and promote immune modulation [97,106,107]. EBV-associated signaling has also been linked to increased survivin expression and the establishment of an immunosuppressive microenvironment that may favor tumor persistence and progression [100]. However, the evidence remains largely associative [103]. There is no consistent evidence that EBV infection precedes tumor development in the prostate, and its detection may reflect viral persistence, reactivation, or permissive conditions within tumor tissue rather than a primary etiological role [108,109]. Therefore, EBV should be interpreted as a potential cofactor rather than an independent etiological agent.

### 5.4. Viral Co-Detection and Potential Cooperation

**Figure 4:**
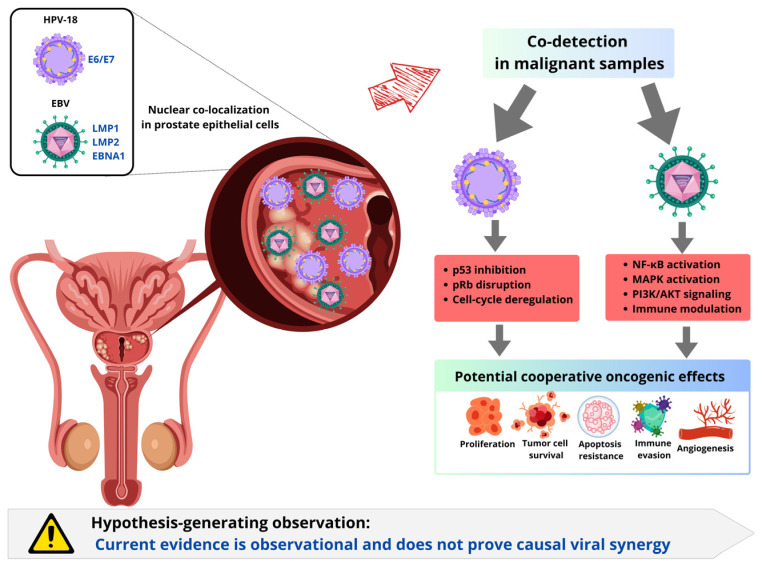
Viral co-detection and potential cooperative oncogenic effects of HPV-18 and EBV in prostate epithelial cells. Abbreviations: HPV, human papillomavirus; HPV-18, human papillomavirus type 18; EBV, Epstein–Barr virus; E6/E7, early viral oncoproteins 6 and 7; LMP1, latent membrane protein 1; LMP2, latent membrane protein 2; EBNA1, Epstein–Barr nuclear antigen 1; p53, tumor protein p53; pRb, retinoblastoma protein; NF-κB, nuclear factor kappa B; MAPK, mitogen-activated protein kinase; PI3K/AKT, phosphoinositide 3-kinase/protein kinase B.

Several studies suggest that multiple oncogenic viruses can be detected in a single prostate cancer sample, implying the possibility of cooperative interactions [97,100]. For example, nuclear colocalization of HPV-18 and EBV has been reported in prostatic epithelial cells, with a significantly higher prevalence of concurrent infection in malignant samples than in benign controls [97]. Such co-detection may be relevant because different viruses can perturb complementary pathways: HPV primarily disrupts p53 and pRb, whereas EBV latent products, including LMP1, LMP2, and EBNA1, have been linked to activation of NF-κB, MAPK, and PI3K/AKT signaling, as well as apoptosis resistance, angiogenesis, survivin upregulation, and immune modulation [97,109,110]. Together, these effects may favor proliferation, tumor cell survival, immune evasion, and resistance to apoptosis, supporting the biological plausibility of viral cooperation within a broader microbial consortium [100], Figure 4. However, current evidence for viral synergy in prostate cancer remains preliminary. Most available data are observational and do not establish whether coinfection has a causal, synergistic, or merely coincidental relationship with malignant transformation. Therefore, viral co-detection should be regarded as a hypothesis-generating observation rather than definitive evidence of cooperative oncogenesis [103].

## 6. The Gut–Prostate Axis as a Systemic Extension of the Microbial Consortium

Remote pathological regulation through the gut–prostate axis is primarily mediated by the systemic circulation of microbial metabolites and the translocation of immune cells [111]. This metabolic crosstalk relies on intestinal microbiota fermentation products, specifically short-chain fatty acids (SCFAs), such as acetate, propionate, and butyrate, derived from the bacterial fermentation of dietary fibers. While SCFAs are typically associated with anti-inflammatory and pro-apoptotic protective mechanisms in the context of colorectal cancer [112], their influence on prostatic oncogenesis appears paradoxically divergent and highly dependent on the specific tissue microenvironment [113].

A mechanistic link has been established a mechanistic link between gut-derived SCFAs and prostatic tumorigenesis, observing that patients with aggressive clinical phenotypes harbor an increased abundance of SCFA-producing bacteria, specifically *Rikenellaceae*, *Alistipes*, and *Lachnospira* [114]. This pro-oncogenic role contrasts with the well-documented anti-inflammatory and protective effects of SCFAs in the gastrointestinal tract [114,115].

The proposed mechanism involves the systemic and local stimulation of Insulin-like Growth Factor-1 (IGF-1); once IGF-1 binds to its receptor (IGF-1R) on prostate epithelial cells, it activates the PI3K/AKT and MAPK/ERK signaling cascades, which function as fundamental drivers of cellular proliferation and survival. Consequently, intestinal dysbiosis modulates oncogenic risk by increasing systemic IGF-1 bioavailability, thereby sensitizing the prostate to mitogenic stimuli and potentially synergizing with androgen-driven growth programs [116].

Beyond inflammation, diet-driven gut dysbiosis may also reprogram prostate cancer metabolism. High-fat dietary patterns enrich microbial functions linked to short-chain fatty acid production, lipid handling, bile acid and cholesterol metabolism, and, in some contexts, steroidogenic capacity [116,117,118]. These metabolites can enhance systemic IGF-1 signaling and activate prostate PI3K/AKT and MAPK/ERK pathways, thereby promoting anabolic growth, survival, and metabolic rewiring [114,116]. Experimental evidence further suggests that gut microbiota-derived metabolites may support autophagy, M2 macrophage polarization, and endocrine resistance, linking dietary fat exposure not only to inflammatory priming but also to tumor cell metabolic adaptation [12,16,117,118]. In parallel, disruption of the intestinal barrier may further amplify this process by facilitating systemic exposure to microbial products such as LPS [111,116]. Together, these findings underscore that the biological effects of microbial metabolites are highly context-dependent and shaped by the receptor and signaling landscape of the target tissue, rather than being uniformly beneficial or harmful [116].

The intestinal epithelial barrier serves as a critical biological checkpoint, sequestering microbial antigens from the systemic circulation. When this barrier is compromised by dysbiosis, frequently a consequence of high-fat Western dietary patterns, increased permeability facilitates the translocation of Pathogen-Associated Molecular Patterns (PAMPs), most notably Gram-negative bacterial LPS, into the bloodstream [111]. Upon reaching the prostate via the vasculature, these endotoxins ligate Toll-like Receptor 4 (TLR4) expressed on both epithelial and resident immune cells [116]. This interaction instigates the canonical NF-kB signaling pathway, driving the transcriptional upregulation of key pro-inflammatory cytokines, including TNF-alpha, IFN-gamma, and IL-6 [115]. This sustained inflammatory state establishes a permissive, pro-oncogenic niche within the prostatic tissue. Chronic TLR4 signaling further promotes malignant progression by stimulating aberrant cellular proliferation and abrogating apoptotic responses, thereby contributing to both the initiation and promotion phases of carcinogenesis [119]. Understanding this bidirectional communication may help explain interindividual variability in disease progression and treatment response, positioning the gut microbiome as a relevant systemic component of the prostate cancer microenvironment.

## 7. Translational Perspectives

Evidence of a prostate–gut–urinary axis necessitates a re-evaluation of current preventive and supportive strategies beyond classical hormonal and lifestyle paradigms. From a preventive standpoint, several non-mutually exclusive strategies can be envisioned.

### 7.1. Microbiome-Targeted Interventions

The identification of microbial and genetic biomarkers offers opportunities for risk stratification and early intervention. Studies of the urinary and tissue microbiome have identified genera such as *C. acnes*, *Mycoplasma* sp., and Cytomegalovirus, among others, present in PCa and associated with inflammation and adverse outcomes [120,121]. These findings suggest that antimicrobial, antiviral, or other targeted strategies that avoid inducing dysbiosis may help modulate the prostatic microenvironment and merit evaluation in rigorously designed clinical trials.

Preclinical evidence does not uniformly support the notion that antibiotic-induced depletion of the gut microbiota delays castration-resistant prostate cancer (CRPC). While some models report delayed progression following the loss of androgen-producing bacteria [12,122], others show accelerated disease progression and reduced ADT efficacy [16,123]. These discrepant findings indicate that the effects of microbiota depletion are highly context-dependent, varying according to the antibiotic regimen, tumor model, and baseline microbial composition. Accordingly, any such claim should be presented as model-specific and still contested, rather than as a universal principle.

Microbiome-aware lifestyle interventions are supported by converging data linking Western dietary patterns, obesity, and dysbiosis to systemic inflammation and PCa risk. Diets enriched in plant-based fibers, omega-3 fatty acids, and polyphenols promote SCFA-producing bacteria and anti-inflammatory profiles, although in PCa, the impact of SCFAs may be context-dependent [117]. Nonetheless, observational and mechanistic studies suggest that dietary modulation that reduces intestinal permeability and endotoxemia (LPS/TLR4 activation) may attenuate the chronic inflammatory tone that primes the prostate for tumorigenesis [14,120].

Similar microbiome-modulating strategies aim to reshape the gut ecosystem toward a less androgenogenic and less inflammatory profile, potentially improving therapeutic responsiveness and delaying disease progression [9]. In other tumor types, fecal microbiota transplantation (FMT) has been associated with improved responses to immune checkpoint inhibitors (ICIs) [124]. Selected commensals and probiotic consortia have likewise been reported to enhance antitumor immune responses in some translational and clinical settings outside prostate cancer [125]. Although primary prevention trials are lacking, these findings support further evaluation of microbiome-targeted strategies, including probiotics, prebiotics, synbiotics, and precisely timed antibiotics or FMT, as adjunctive approaches to reduce progression risk in men with high-grade prostatic intraepithelial neoplasia, chronic prostatitis, or early PCa under active surveillance [12,124].

### 7.2. Microbiome and Treatment Resistance

Microbiome-aware supportive strategies should also account for therapy-induced dysbiosis as both a potential risk factor and a modifiable target. Androgen deprivation therapy and next-generation androgen axis inhibitors can reshape the gut microbiota, favoring the expansion of commensal species capable of de novo androgen synthesis, which may contribute to endocrine resistance. Radiotherapy and systemic treatments may further disrupt microbial balance by depleting beneficial commensals and exacerbating mucosal inflammation [12,120]. In this context, preventive approaches could include baseline microbiome profiling, minimizing unnecessary use of broad-spectrum antibiotics, and early implementation of microbiome-supportive measures in patients initiating long-term androgen axis therapies [13,126]. Such strategies aim to preserve microbial diversity, reduce inflammation, and potentially limit microbiome-driven mechanisms of treatment resistance [14,127]. However, these interventions remain investigational, and well-designed randomized clinical trials are needed to determine which microbiome-modulating approaches can be safely and effectively integrated into prostate cancer prevention, treatment optimization, and survivorship care [15,16].

## 8. Limitations

This review has several limitations. First, although the literature search was broad and up to date, it was not conducted or reported in accordance with a formal systematic review or PRISMA-ScR framework, which may have led to selection bias and an incomplete capture of relevant studies. The review was conceived as a narrative, hypothesis-generating synthesis; therefore, no meta-analysis or formal risk-of-bias assessment was performed. Second, the field of prostate microbiome research is dominated by low-biomass samples and heterogeneous sequencing pipelines, making it difficult to distinguish true intraprostatic colonization from contamination and to compare taxa across studies. These factors make it difficult to distinguish true intraprostatic microbial signals from contamination and to compare findings across studies. Third, much of the mechanistic evidence regarding microbial signaling pathways derives from non-prostate models, including gastric, colorectal, cervical, and broader host–microbe systems. As a result, extrapolation of these mechanisms to prostate cancer should be made with caution.

Importantly, most currently available human data are associative rather than causal. Although multiple bacterial and viral taxa have been detected in prostate-related specimens and linked to inflammatory, metabolic, or oncogenic signaling pathways, there is still no consistent longitudinal or interventional evidence demonstrating that these microbial consortia directly initiate or drive prostate carcinogenesis. Accordingly, many of the relationships discussed in this review should be interpreted as hypothesis-generating rather than definitive evidence of causation. Finally, both the benign prostatic hyperplasia and prostate cancer microbiome literature remain limited by small cohorts, methodological inconsistency, and variable specimen sources, including tissue, urine, semen, and expressed prostatic secretions. These differences complicate direct comparisons between benign and malignant states and limit the ability to define stable disease-specific microbial signatures. Future studies will require standardized sampling, rigorous contamination control, longitudinal design, and integrated multi-omic validation in prostate-specific models.

## 9. Conclusions

Current evidence supports a model in which prostate carcinogenesis may be influenced by sustained interactions between host biology and dynamic microbial genomic consortia operating across local and systemic compartments. Rather than functioning as isolated infectious agents, bacteria and viruses associated with the prostate appear to converge on shared inflammatory, metabolic, and oncogenic signaling networks that shape the tumor microenvironment and may contribute to disease initiation, progression, and treatment response. The emerging concept of a prostate–gut–urinary axis further expands this framework, suggesting that distal microbial communities can modulate prostatic physiology through endocrine, immune, and metabolic pathways.

However, the available evidence remains predominantly correlative, and the causal contribution of microbial consortia to prostate carcinogenesis has not yet been established. Technical challenges inherent to low-biomass microbiome research, together with methodological heterogeneity and the frequent need to extrapolate from non-prostate models, warrant cautious interpretation of current findings.

Future research should prioritize rigorous contamination control, longitudinal sampling, standardized analytical pipelines, multi-omic integration, and mechanistic validation in prostate-specific systems. If confirmed, microbial signatures and microbiome-associated pathways may offer new opportunities for biomarker development, risk stratification, and targeted modulation of the tumor microenvironment. Collectively, these findings support an expanded ecological view of prostate cancer in which microbial consortia should be regarded as potential modulators of tumor biology rather than definitively established drivers.

## Figures and Tables

**Table 1:** Comparative features of the stromal, immune, microbial, and regulatory micro-niche in benign prostatic hyperplasia and prostate cancer.

Micro-Niche Feature	Benign Prostatic Hyperplasia (BPH)	Prostate Cancer
Stromal composition	Stroma remains enriched in differentiated smooth muscle cells and normal fibroblasts, preserving tissue architecture and homeostatic epithelial support [55].	Progressive smooth muscle depletion accompanies the emergence of CAFs, which remodel the extracellular matrix and promote invasion and metastatic dissemination [55].
Stromal-epithelial signaling	Stromal cells express canonical and noncanonical Wnt ligands, including Wnt5a, which restrain epithelial proliferation and maintain relative growth quiescence [56].	Homeostatic stromal–epithelial signaling is disrupted; stromal AR-mediated paracrine cues shift toward growth-promoting programs that support malignant epithelial expansion [57].
Immune microenvironment	Aging-related androgen decline is associated with a mildly activated immune environment, including a reduced Treg/CD4+ T-cell ratio and increased granzyme expression, which may favor vascular growth and fibrotic remodeling [58].	The tumor microenvironment is typically immunosuppressive; stromal fibroblasts and other niche components express mediators such as TGF-β, IDO, and PD-L1, thereby limiting cytotoxic T-cell activity and facilitating immune evasion [59].
Single-cell immune profile	Peripheral blood mononuclear cells show monocytes enriched in cholesterol-storage and Notch-signaling pathways, with cell–cell communication involving MIF- and galectin-related interactions [60].	PCa is associated with increased CD14+ monocytes, NK cells, and γδ T cells; monocytes show enrichment of tumor-progression-associated markers and interleukin-27 signaling, with TGF-β-dominated intercellular communication [60].
Local microbial landscape	Benign tissue is associated with a distinct commensal profile, including *Kocuria palustris* and *Cellvibrio mixtus*; other local findings include *Streptococcus mitis*, *Staphylococcus haemolyticus*, *Chlamydia trachomatis*, and *Cutibacterium acnes* [54].	Malignant tissue is enriched for taxa such as *Cupriavidus taiwanensis*, *Methylobacterium organophilum*, *Escherichia coli*, *Fusobacterium nucleatum*, and *Cutibacterium acnes*, *Shewanella*, *V. parahaemolyticus*, *Microbacterium* sp., among others [46,48,54].
Gut-associated microbial changes	Reported alterations include an increased Firmicutes/Bacteroidetes ratio, enrichment of *Prevotella*, *Ruminococcus*, *Turicibacter*, and *Clostridium*, and reduced *Lactobacillus*; *Escherichia–Shigella* has been linked to BPH risk and LUTS severity [50,52].	Gut and tissue microbial changes in PCa appear more strongly associated with dysbiosis-related inflammatory and tumor-promoting signatures than with benign remodeling alone [50].
Virus-associated findings	No dominant virus-associated niche pattern is emphasized.	Tumor-associated viral signals, including EBV, HBV, HPV-16, and HPV-18, have been linked to PCa and correlate with the PCa-associated bacterial signature [54].
Stromal inductive capacity	Stromal cells combined with BPH-1 epithelial cells generate small, organized, sharply demarcated grafts, indicating controlled inductive potential [61].	Cancer-derived stromal cells combined with BPH-1 cells generate disorganized, invasive grafts that extend into adjacent host tissue, consistent with an aggressive phenotype [61].
Reactive stroma and CAFs	Stroma remains largely composed of differentiated smooth muscle and fibroblasts that support organ homeostasis through balanced growth-factor and cytokine secretion [62].	Reactive stroma with phenotypically altered CAFs secretes growth factors, cytokines, and matrix-remodeling enzymes that sustain cancer stemness and therapy resistance [63].
Androgen receptor niche	Stromal AR supports epithelial–stromal crosstalk and contributes to prolactin-driven hyperplastic signaling through GM-CSF/STAT3-related pathways [64].	Although AR-deficient mesenchyme cannot support normal prostatic development, aberrant stromal AR activity in established tumors promotes malignant progression and may represent a therapeutic target [65].
Aging-related stromal changes	Aging stroma exhibits inflammatory, oxidative, and matrix-disorganizing changes, with increased macrophage and T-cell infiltrates that may prime the tissue microenvironment for disease [66].	Tumor-adjacent stroma shows bone-remodeling and immune-related transcriptional programs that distinguish aggressive from indolent disease and may predict metastatic progression [67].

Abbreviations: AR, androgen receptor; BPH, benign prostatic hyperplasia; CAFs, cancer-associated fibroblasts; CD4+, cluster of differentiation 4-positive T cells; CD14+, cluster of differentiation 14-positive monocytes; EBV, Epstein–Barr virus; GM-CSF, granulocyte–macrophage colony-stimulating factor; HBV, hepatitis B virus; HPV-16, human papillomavirus type 16; HPV-18, human papillomavirus type 18; IDO, indoleamine 2,3-dioxygenase; LUTS, lower urinary tract symptoms; MIF, macrophage migration inhibitory factor; NK, natural killer cells; PCa, prostate cancer; PD-L1, programmed death-ligand 1; TGF-β, transforming growth factor beta; Treg, regulatory T cells; Wnt, Wingless/Integrated signaling pathway.

**Table 2:** Prostate-associated microorganism and viruses: specimen type, detection method, principal signaling pathways, tumor-relevant phenotypes, and type of supporting evidence.

Pathogen		Specimen Type	Detection Method	Key Virulence Factor(s)	Principal Signaling Pathway(s)	Tumor-Relevant Phenotype	Type of Supporting Evidence
Bacterial members	*Cutibacterium acnes* [81,82]	Tissue, macrophages	MLST, 16S, Culture	Peptidoglycan (PGN), CAMP1 protein	TLR2/TLR4 → NF-κB; MAPK; cGAS–STING	Chronic inflammation, IL-6/CXCL8 production, proliferative microenvironment	Human tissue detection; mechanistic prostate model
	*Escherichia coli* [83]	Urine, Tissue, CA	16S, High-sensitivity Seq	Lipopolysaccharide (LPS), Cytotoxic necrotizing factor-1 (CNF1)	TLR4 → NF-κB; IL-6/STAT3; AKT/GSK-3β/β-catenin; Cdc42–PAK1 axis	Inflammation, matrix degradation, invasion, metastasis	Mechanistic prostate model; extrapolated evidence
	*Pseudomonas aeruginosa* [84]	Tissue, Urine	mNGS, FISH, qPCR	LPS	TLR4 → NF-κB (MyD88-dependent)	Increased proliferation, reduced apoptosis	Human tissue detection; extrapolated evidence
	*Porphyromonas gingivalis* [85]	Tissue, Swab	16S, qPCR, IHC	PGN	NOD1/NOD2 → RIP2 → NF-κB and MAPK (JNK)	PD-L1 upregulation, immune evasion	Human tissue detection; extrapolated evidence
	*Fusobacterium nucleatum* [36]	Tissue, Midstream Urine	16S, qPCR	Fap2 protein	TIGIT receptor interaction	Protection from NK-cell-mediated cytotoxicity, immune evasion	Human tissue detection; extrapolated evidence
	*Mycoplasma* spp. [86,87,88]	Tissue, Urine, Semen	PCR, qPCR, Culture	p37 membrane lipoprotein	EGFR → PI3K/AKT; PKC; MAPK/RAS	Enhanced survival, proliferation, and invasion	Human tissue detection; mechanistic prostate model
	*Chlamydia trachomatis* [81,82,89]	Tissue, Semen, EPS	LCR, NAAT, ELISA	LPS, HSP60, MIP-like protein	NF-κB activation; TLR2/TLR4 upregulation	Proliferation, angiogenesis, metastasis, therapeutic resistance	Human tissue detection; extrapolated evidence
	*Helicobacter pylori* [26,90]	Tissue (Malignant)	PathoChip, PCR, IHC	CagA, VacA, LPS (proposed)	NF-κB; IL-6/gp130 → JAK/STAT3	Chronic inflammation, tumor-promoting signaling	Human tissue detection; extrapolated evidence
Viral members	Human papillomavirus (HPV) [91,92,93]	Tissue, Exosomes, Urine	Nested PCR, NGS, ISH	E6/E7 oncoproteins	p53 degradation (E6); pRb inactivation (E7); NF-κB and survival signaling	Loss of cell-cycle control, genomic instability, apoptosis resistance	Human tissue detection; extrapolated evidence
	Cytomegalovirus (HCMV) [44,84]	Tissue (PIN/lesions)	IHC, ISH, PCR, Seq	IE1/IE2, UL38, UL133–UL138, US28	PI3K/AKT/mTOR; NF-κB; JAK/STAT; c-Myc	Oncomodulation, proliferation, survival, androgen-axis interaction	Human tissue detection; mechanistic prostate model; extrapolated evidence

Abbreviations: AKT, protein kinase B; CA, *Corpora amylacea*; CAMP1, Christie–Atkins–Munch-Petersen factor 1; cGAS, cyclic GMP–AMP synthase; CNF1, cytotoxic necrotizing factor 1; CXCL8, C-X-C motif chemokine ligand 8; EGFR, epidermal growth factor receptor; ELISA, enzyme-linked immunosorbent assay; EPS, expressed prostatic secretion; FISH, fluorescence in situ hybridization; HSP60, heat shock protein 60; IHC, immunohistochemistry; IL-6, interleukin-6; ISH, in situ hybridization; JAK, Janus kinase; JNK, c-Jun N-terminal kinase; LCR, ligase chain reaction; LPS, lipopolysaccharide; MAPK, mitogen-activated protein kinase; MIP, macrophage infectivity potentiator; MLST, multilocus sequence typing; mNGS, metagenomic next-generation sequencing; mTOR, mechanistic target of rapamycin; NAAT, nucleic acid amplification test; NF-κB, nuclear factor kappa B; NGS, next-generation sequencing; NK, natural killer; NOD, nucleotide-binding oligomerization domain; p53, tumor protein p53; pRb, retinoblastoma protein; PCR, polymerase chain reaction; PGN, peptidoglycan; PI3K, phosphoinositide 3-kinase; PKC, protein kinase C; qPCR, quantitative polymerase chain reaction; RIP2, receptor-interacting protein kinase 2; Seq, sequencing; STING, stimulator of interferon genes; STAT3, signal transducer and activator of transcription 3; TLR, Toll-like receptor.

## Data Availability

Data sharing is not applicable to this article, as no new datasets were generated or analyzed. All information is derived from previously published studies cited in the reference list.

## Abbreviations

ADT	Androgen deprivation therapy
AKT	Protein kinase B
BPH	Benign prostatic hyperplasia
cGAS	Cyclic GMP–AMP synthase
CRPC	Castration-resistant prostate cancer
EBV	Epstein–Barr virus
EMT	Epithelial–mesenchymal transition
ERK	Extracellular signal-regulated kinase
FMT	Fecal microbiota transplantation
HCMV	Human cytomegalovirus
HPV	Human papillomavirus
IGF-1	Insulin-like growth factor 1
IL	Interleukin
JNK	c-Jun N-terminal kinase
MAPK	Mitogen-activated protein kinase
mTOR	Mechanistic target of rapamycin
NF-κB	Nuclear factor kappa B
PAMP	Pathogen-associated molecular pattern
PCa	Prostate cancer
PGN	Peptidoglycan
PI3K	Phosphoinositide 3-kinase
STING	Stimulator of interferon genes
TLR	Toll-like receptor
